# Editorial: Application of systems biology in molecular characterization and diagnosis of cancer, Volume II

**DOI:** 10.3389/fmolb.2022.1089463

**Published:** 2022-11-25

**Authors:** Yongjun Wei, Cheng Zhang, Adil Mardinoglu, Peng Zhang

**Affiliations:** ^1^ School of Pharmaceutical Sciences, Laboratory of Synthetic Biology, Zhengzhou University, Zhengzhou, China; ^2^ Science for Life Laboratory, KTH—Royal Institute of Technology, Stockholm, Sweden; ^3^ Faculty of Dentistry Oral and Craniofacial Sciences Centre for Host-Microbiome Interactions, King’s College London, London, United Kingdom; ^4^ Beijing Pediatric Research Institute, Beijing Children’s Hospital, Capital Medical University, National Center for Children’s Health, Beijing, China

**Keywords:** cancer, systems biology, precision medicine, multi-omics, molecular subtype

Cancer is the leading death cause worldwide, and it is one of the biggest contributors for premature mortality (Bray et al., 2021). According to the global cancer statistics in 2020, 19.3 million new cancer cases were identified, and 10 million cancer patients were died (Sung et al., 2021). Normally, due to the heterogeneity in individual tumor, many cancer patients have distinct and diverse cancer cells, and are resistant to current drug therapies (Dagogo-Jack and Shaw, 2018). Thus, understanding molecular mechanisms and influencing factors during cancer incidence and development might provide effective prevent, diagnosis, and treatment strategies (Elmore et al., 2021). Systems biology can give insight into the complex biological systems and provide promising tools to recover underlying cancer molecular characterization, which would help establish personalized precise cancer diagnosis and therapy strategies (Shi et al., 2020). Therefore, application of systems biology in molecular characterization and diagnosis of cancer is of great interest.

Breast cancer was the most commonly diagnosed cancers in 2020, and about 2.3 million new breast cancer cases were diagnosed, which composed 11.7% of all the diagnosed cancer. Breast cancer was the fifth leading cause of cancer death, and 684,996 breast cancer patients were died in 2020 (Sung et al., 2021). Liu et al. developed a novel YTHDF3-based model *via* analyzing expression profiles derived from the cancer genome atlas. This model can be used to predict the overall survival of breast cancer patients and evaluate the treatment of current therapeutic agents for breast cancer patients. Therefore, this novel model would improve the therapeutic effects for breast cancer patients in the future. Asim et al. revealed the underlined mechanism of one epigenetic tumor suppressor gene of Runt-related transcription factor 3 (RUNX3) using formal model and machine learning strategies. Moreover, they identified a potential epigenetic drug target of DNA methyltransferase 1 (DNMT1) for breast cancer patients.

The development of high-throughput sequencing generates abundant multi-omics data of cancer patients, and provides the opportunities to develop effective diagnosis and prognosis strategies for cancers. Gao et al. identified key hub genes in the development and progression of hepatocellular carcinoma using bioinformatic analysis and established a prognostic model. They further verified these key genes and the model using International Cancer Genome Consortium (ICGC) dataset. This might bring effective hepatocellular carcinoma prediction and diagnosis methods for the patients. Guo et al. identified eight necroptosis-related genes related with the glioma immune microenvironment *via* single-cell and bulk RNA sequencing data. This might be used to predict the prognosis of glioma and provide precise glioma investigation. Fan et al. found that ferroptosis and immunity might participate in the progression of thyroid carcinoma. These results had potential application in prediction of the prognosis and clinical treatment of thyroid carcinoma.

The application of systems biology can recover potential prediction, diagnosis, and therapy treatment strategies for diverse cancers, which would lead to design personalized and precise cancer treatments. As the cancer data are big, machine learning and other artificial intelligence methods should be introduced to investigate cancer diseases (Elemento et al., 2021). Moreover, gut microbiota is associated with diverse diseases and has effects on drug effectiveness (Yang et al., 2021; Sugimura et al., 2022; Yang et al., 2022). Thus, recovering the causality between gut microbiota and cancer and engineering gut microbiota of cancer patients would contribute to future cancer treatments (Zhou et al., 2021).
